# A geometric approach for accelerating neural networks designed for classification problems

**DOI:** 10.1038/s41598-024-68172-6

**Published:** 2024-07-30

**Authors:** Mohsen Saffar, Ahmad Kalhor, Ali Habibnia

**Affiliations:** 1 School of Electrical and Computer Engineering, College of Engineering, University of Tehran, Tehran, Iran; 2 Department of Economics and the Computational Modeling and Data Analytics, College of Science, Virginia Polytechnic Institute and State University, Blacksburg, USA

**Keywords:** Network compression, Convolutional neural network, Dataflow evaluation, Separation index, Computer science, Electrical and electronic engineering

## Abstract

This paper proposes a geometric-based technique for compressing convolutional neural networks to accelerate computations and improve generalization by eliminating non-informative components. The technique utilizes a geometric index called separation index to evaluate the functionality of network elements such as layers and filters. By applying this index along with center-based separation index, a systematic algorithm is proposed that optimally compresses convolutional and fully connected layers. The algorithm excludes layers with low performance, selects the best subset of filters in the filtering layers, and tunes the parameters of fully connected layers using center-based separation index. An illustrative example of classifying CIFAR-10 dataset is presented to explain the algorithm step-by-step. The proposed method achieves impressive pruning results on networks trained by CIFAR-10 and ImageNet datasets, with 87.5%, 77.6%, and 78.8% of VGG16, GoogLeNet, and DenseNet parameters pruned, respectively. Comparisons with state-of-the-art works are provided to demonstrate the effectiveness of the proposed method.

## Introduction

Recently, Convolutional Neural Networks (CNNs) are applied to solve challenging decision-making problems dealing with high-dimensional and large datasets^1^. CNNs achieve promising results and improved accuracy compared to the typical neural networks thanks to their structure containing a sequence of convolutional layers (CLs) and billions of trainable parameters^2^. High-tech GPUs with the massive capability of computations are needed for tuning CNNs’ parameters to achieve the maximum performance of these networks. Besides, recalling such a huge network is time-consuming, even with a top-notch hardware system. It should be mentioned that when it comes to real-world scenarios, especially with real-time purposes, such time for training or recalling a network is intolerable.

Another concern for storing the parameters related to these bulky networks is the memory which can get more serious when it comes to portable devices with limited resources and bandwidths^3^. In recent years, the market of some mobile devices such as smart wearable, virtual and mixed reality-based devices have been growing to an increasing extent. These products take advantage of Artificial Intelligence (AI) algorithms with limitations in storing and computation. CNNs as AI structures should be more compact to be deployable in those small-size products. Fortunately, the literature demonstrates that some modifications can be made to optimize and speed-up CNNs. Some of them can be applied to a pre-trained model and accelerate the forward recall process, while some other methods compact the model’s structure and the parameters needed to be trained from scratch. In the following, many approaches in this area will be considered, and their strengths and weaknesses will be discussed.

By neglecting the least significant bits of parameters' values, their numerical resolution can be decreased to free up some memories^4^. In^5^, Huffman coding is also applied along with the quantization, and more occupied memories become available. In an extreme case, the parameters' value can even be represented in binary-scheme, so only one bit can characterize each weight^6^. Tuning weights by quantization is efficient and straightforward, while performance decreases when CNN layers grow.

Considering the complex and large structure of CNNs, the existence of unnecessary parameters does not seem unlikely. Denil et al.^7^ shows that only a tiny group of weights is enough to rebuild the whole network. Thus, in ^8^, writers define a hash function that gathers similar weights into some groups. After that, each group called hashed bucket is replaced with a single weight. Moreover, a conventional backpropagation algorithm is also applicable for training the HashedNets.

In^9^, the authors concentrate on the redundancy existing in Fully Connected Networks (FCLs). The operation in these layers can be formulated as $f\left(x\right)=\sigma (Ax)$, where $\sigma (.)$ is a nonlinear map, $x$ is the input vector, and $A$ is the weight matrix. They provide a more compact alternative to the conventional dense matrix $(A),$ which occupies less memory. The proposed matrix eases the training process using fast matrix–vector multiplication. Besides FCLs, the structural matrix can be used even in multi-dimensional CNNs. The severity of such work is that there is no predetermined template for the structural matrix.

Another solution is proposed for eliminating the redundancies in CNN layers in^10^. The authors introduce 1-rank basis filters, which can reconstruct the original convolutional 2D and 3D filter banks. These low-rank filters are trained so that the new network mimics the input–output mapping of the primary network. To this end, two optimization techniques are defined for training new weights. It should be noted that the approximation process is taken to account layer by layer. Thus, a suboptimal solution may be achieved in this manner. Additionally, it takes time to attain each layer convergence, and the problem worsens when the network has many CNN layers. Moreover, this method cannot address clearly how over-fitted conditions can be handled in the compressed network.

Some works indicate that a bit of modification in standard RELU can either produce or discover new informative features. After the training process has been done, some observations indicate that each filter has its own pairing filter with a negative phase^11^. A new RELU-like activation that does not zero out negative magnitude is proposed to maintain both positive and negative information^12^. A multi-bias RELU is another modified RELU that generates multi feature maps considering the magnitude of the input^13^. In addition to the sign and magnitude of input, rotation and filliping operators are also considered in^14^ to generate multiple features from a single feature map. Thanks to these modified RELUs, the CNN structure can be implemented with fewer filters and parameters without reducing performance. Nonetheless, the amendments above are application-based and cannot be assume as general compressing law to be implemented in every network with every dataset.

Knowledge Distillation (KD) is another method to extract a compact net from the original one. Notably, the compact network should react like the original network, but it is not trained only to imitate the class labels. The authors in^15^ mentioned that SoftMax output for each data could be considered significant decoded information. Therefore, in an optimization procedure, the distilled net is forced to reproduce both the class labels and probabilities generated by the SoftMax function of the original network located at the end of FCLs. An extended work in this area is^16^, in which the compact network tries to reproduce some of the feature maps in addition to the SoftMax probabilities. Even online training is investigated for deriving student models^17^. One disadvantage of the KD model is that the method is only deployable for SoftMax-based structures.

Network pruning as an effective method can compress the network and improve generalization by eliminating redundant connections or elements^18–35^. Pruning techniques can be utilized on pre-trained models or those trained from the scratch, typically categorized into two main types based on the extent of pruning: unstructured and structured pruning.

Unstructured pruning involves masking redundant, low-weight connections between network layers. For instance, in^22^, a pruning method is established wherein smaller weights are removed compared to a threshold computed based on the standard deviation of the corresponding layer. In addition, the redundancy can be captured using some constraints on convolutional filters such as algebraic norms. For instance, in^25^, ${l}_{1}$ norm is utilized in a regularization process to eliminate weights in a group-wise manner. However, convergence with l-norm regularization tends to be prolonged, necessitating numerous iterations. Moreover, determining the pruning threshold often requires manual tuning, which can be cumbersome in certain scenarios. It is worth noting that this type of pruning can be applied either after training^36^ or during training, with the former typically being more successful in maintaining network performance but requiring more time. The irregular sparsity patterns resulting from such unstructured pruning methods necessitate the use of specialized software and hardware for efficient management.

In contrast, structured pruning eliminates entire structures within the network, such as channels, filters, or layers, resulting in more hardware-friendly sparse models. This method ensures that the pruned model maintains a regular structure, facilitating more straightforward acceleration on common hardware^37^. He et al.^38^ prunes neurons by utilizing their activation entropy. Additional fine-tuning is also needed since this method affects the performance of the network. Alqahtani et al.^39^ ranks the neurons based on the activation values and prunes less effective ones. Sometimes, there exist similar neurons in a structure. Srinivas and Babu^40^ introduced a method to detect similarity in neurons and remove other alternatives while^41^ merge the similar neurons into one. By ranking the elements at filter level, one can prune the less important filters. The contribution of each filter is evaluated by measuring different approaches, e.g. variance of channel^42^, mean gradient of feature maps or entropy of channel outputs^43^. Furthermore, structured pruning methods also can be implemented in layer level^44,45^, offering faster processing but potentially compromising network performance compared to connection pruning methods, often necessitating retraining phases to achieve optimal results.

In the pruning methods and indirectly in other compressing methods, an evaluating algorithm is needed to measure the performance of different parts in the network. Such an algorithm can detect which network component is effective or redundant. Cost-functions are well-known evaluation functions that measure a network's performance at the final layer^46^. However, for compressing purposes, an evaluation task must be implemented for each layer or even each filter since the location of redundancies is unknown.

In our previous work^47^, we have introduced the Separation Index (SI) as a complexity measurement that is capable of evaluating different parts of the network in classification problems. Building upon that research, we have now investigated that SI can bring to light the redundancy by evaluating different parts of the network. Furthermore, the SI has found applications in diverse fields, underscoring its efficacy in assessing different network layers.

The SI is also used to perform identification based on hand and face biometrics^48^. Initially, the SI ranks various state-of-the-art deep convolutional neural networks based on their accuracy in the identification task. Then, as a complexity measure, the SI evaluates the last layer of the best-performing network and compresses it by removing neurons that produce ineffective features. In^49^, an attention mechanism is introduced based on the SI to weigh different feature maps generated by a modified EfficientNet-b7. These feature maps are used for a video-based person re-identification task. Additionally, extensions of the SI, such as the Smoothness Index (SmI), have been employed for regression problems, as showcased in^50^, where they improve the performance of feature selection methods.

In this paper, we introduce a novel pruning compression method based on the Separation Index, which effectively measures the significance of connections or elements within the network, such as filters, neurons, or layers. Our method initially removes less critical layers and subsequently identifies and prunes redundant filters among the remaining structures. This technique eliminates the necessity for complete retraining and preserves the performance of the original network. Our proposed method offers the following features:

- Eliminates the need for training from scratch, while still achieving comparable or superior results to methods requiring retraining.
- Identifies redundancy at the neuron, channel, or layer level, boosting the compression process, particularly in its early stages.
- Applicable to both convolutional and fully connected layers, wherever redundancies are present.
- Introduces a novel approach to designing fully connected structures with fewer parameters, preserving the generalization achieved by filtering layers.
- Simple to learn and implement.

## Evaluation of dataflow in classification problems

### Convolutional neural network architecture for classification

In classification problems, the goal is to train a classifier to associate input patterns $({\left\{{x}_{0}^{q}\right\}}_{q=1}^{Q})$ with their respective classes $({\left\{ {c}^{q}\right\}}_{q=1}^{Q})$. The CNN structure with ${n}_{L}$ layers is assumed to be the classifier model. During the training process, the network parameters are adjusted by feeding $Q$ input data $\{{x}_{0}^{q}{\}}_{q=1}^{\text{Q}}$ to the classifier and obtaining output values that aim to imitate the target values $\{{c}^{q}{\}}_{q=1}^{Q}$ where ${c}^{q}\epsilon \left\{\text{1,2},\dots ,{n}_{C}\right\}$ and ${n}_{C}$ refers to number of classes.

From a geometric viewpoint, convolutional filters eliminate redundant and noisy elements from the raw input data to construct separable sub-patterns from an initial unorganized dataset. These sub-patterns are gradually formed through a sequence of CLs and many training epochs. Notably, each CL and its set of filters has limited capability to conduct input data to sensible patterns. Therefore, the complexity of raw data is reduced gradually layer by layer. This argument that clusters containing same-label samples are formed gradually through network layers is well-defined in^51^ by Goldfeld. Subsequently, his observation is also confirmed and shared in^52,53^.

### Separation index

The Separation Index formerly introduced in^47^ is originally an complexity measurement tool which can be used to evaluate the feature maps extracted at any layer or filter of convolutional neural network. It is worth noting that the index can be applied to any network involved in classification tasks, not just CNNs. The full description of SI is provided in^47^ and in this paper, only the first order of the SI is discussed. Assume that the output of *l*th layer for all samples ($\{{x}_{0}^{q}{\}}_{q=1}^{\text{Q}}$) known as dataflow at *l*th layer ($\{{x}_{l}^{q}{\}}_{q=1}^{\text{Q}}$) is available. SI is calculated for this dataflow as follows:

1 $$ \begin{aligned} & {\text{SI}}(\{ x_{l}^{q} \}_{q = 1}^{Q} ) = \frac{1}{{\text{Q}}}\mathop \sum \limits_{q = 1}^{Q} \delta \left( {c^{q} - c^{{q^{*} }} } \right), \\ & q^{*} = \mathop {\arg } \mathop {\min }\limits_{{q^{\prime}}} \left\| {x_{l}^{q} - x_{l}^{{q^{\prime}}} } \right\|, \\ & q^{\prime} \in \left\{ {1,2,..,Q} \right\}\, {\text{and}} \,q^{\prime} \ne q, \\ & \delta \left( \upsilon \right) = \left\{ {\begin{array}{*{20}c} 1 & {\upsilon = 0} \\ 0 & {\upsilon \ne 0} \\ \end{array} } \right., \\ \end{aligned} $$

where $\delta (.)$ is delta Kronecker function. Besides, to measure the distance between two arbitrary samples ($\Vert {x}_{l}^{q}-{x}_{l}^{{q}{\prime}}\Vert$), n-dimensional representation of data can be transformed to a reshaped 1-dimensional vector.

The SI indicates the complexity of the raw data and represents the ratio of easy-to-classify samples in a dataset. These samples are not located in overlapped region where the boundaries separate different classes. This approach to measure complexity resembles the approach discussed in problems with intra-class imbalances^54^. As a result, the closer this index gets to its maximum value for a given dataset, the lower the complexity of the data. According to the formulation, the valid range for SI is $[0\, 1]$ interval.

**Figure 1:**
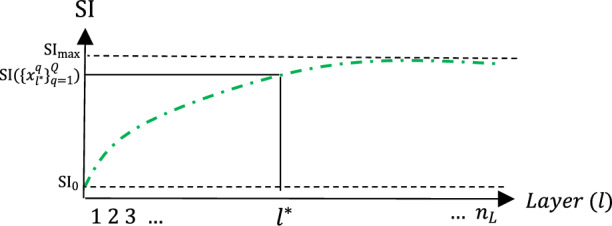
SI increasing behavior across network layers. The ${{l}^{*}}$th location and its corresponding SI value is located.

Investigating and measuring how SI changes through layers can lead to a proper comprehension of the network’s performance in the layer or even filter scale. An almost increasing behavior is expected if SI’s variation trend for all CLs is plotted. This behavior is shown in Fig. 1 as a symbolic graph. If a layer does not contribute in increasing SI, this layer contains redundant and irrelevant filters. In section III, it is shown that how these filters can be captured and set aside in a network compression algorithm.

### Center-based separation index

An extension of SI which has more correlation with accuracy is introduced in this section. In this manner, instead of regarding the nearest neighbor, the distance of each sample to each focal point is noticed. A focal point of *l*th layer and *c*th class is computed as follows:

2 $$ \begin{aligned} & fp_{l,c} = \frac{1}{{Q_{c} }}\mathop \sum \limits_{q = 1}^{Q} x_{l}^{q} \delta \left( {c^{q} - c} \right), \\ & c \in \left\{ {1,2, \ldots ,n_{c} } \right\}, \\ & l \in \left\{ {1,2, \ldots ,n_{L} } \right\}; \\ \end{aligned} $$

where ${Q}_{c}$ is the total number of samples belonging to *c*th class and ${x}_{l,c}^{q}$ is the output of* l*th layer of *q*th sample belonging to *c*th class. As it can be understood, the focal point is the geometrical average of samples with the same label, a proper representation of an approximate class location. Regarding focal point's definition, a new geometric index which is called center-based separation index (CSI), can be calculated as follows:

3 $$ \begin{aligned} & {\text{CSI}}(\{ x_{l}^{q} \}_{q = 1}^{Q} ) = \frac{1}{{\text{Q}}}\mathop \sum \limits_{q = 1}^{Q} \delta \left( {c^{q} - c_{l}^{q} } \right), \\ & c_{l}^{q} = \mathop {\arg }\limits_{{\phantom{0}}} \mathop {\min }\limits_{c^{\prime}} x_{l}^{q} - fp_{l,c^{\prime}} , \\ & c^{\prime} \in \left\{ {1,2,..,n_{c} } \right\}, \\ \end{aligned} $$

CSI measures what proportion of all samples is close to the focal point of its kind. Higher CSI at any layer means that the samples related to each class form circular patterns separated from the focal point of other classes. For example, if a filtered sample belonging to *a*th class is similar to the focal point belonging to *b*th class, there is a high possibility that this sample is not filtered efficiently. Consequently, it could be misclassified as a member of *b*th class in the partitioning part, according to its inappropriate location in feature space.

## Compression with SI

In this section, a new method for compressing and accelerating of CNN based on SI is introduced. However, this method is not limited to CNNs and can be extended and implemented on other structures with few formulation changes. In this method, different parts of the network are evaluated by SI to see whether they are redundant or “effective”. Here, the “effective” element is considered an element that can play a role in SI improvement. Also, a filter or a layer that cannot improve SI is considered an “ineffective” network element. In fact, the improvement in SI is assumed to be equivalent to an improvement in network performance, which seems logical according to the previous section. Besides, in^47^, it is shown that SI and accuracy has a meaningful correlation. Our novel method consists of three main steps described in the following.

### Remove “ineffective” CLs

In a well-trained CNN, there is a possibility that some last CLs are not effective in terms of improving classification rate. It happens when there are more layers than is actually needed. For example, in transfer learning scenarios, sometimes the chosen pre-trained network is too sophisticated for the desired data. This over-designed situation can be detected by monitoring SI value through layers according to its mentioned relevance with accuracy. Figure 1 indicates SI value for different layers of a sample trained CNN. As it can be seen, the value does not improve from a threshold after a certain number of layers. Those layers that do not improve SI may even adversely affect network generalization. Therefore, to have a more compressed network with a better generalization, these layers can be eliminated without losing performance. It should be noted that the SI of remaining CLs should not decrease in proportion to $S{I}_{max}=\underset{l}{\text{max}}SI(\{{x}_{l}^{q}{\}}_{q=1}^{Q})$. Therefore, the smallest $l$ $({l}^{*})$ that satisfied the following inequality can be chosen, and any further CL will be removed in the compressing process.

4 $$ \begin{aligned} & \frac{{{\text{SI}}_{max} - {\text{SI}}(\{ x_{l}^{q} \}_{q = 1}^{Q} )}}{{{\text{SI}}_{max} }}*100 \le P_{l} \% , \\ & l \in \left\{ {1,2, \ldots ,n_{L} } \right\}; \\ \end{aligned} $$

where ${n}_{L}$ indicates the last CL of the original network. ${P}_{l}$ is the percentage error that can be tolerated in the “ineffective” layer removing process. Due to maintaining the classifier performance, ${P}_{l}$ can be chosen up to 2%. In section V.B, the variation effect of this parameter is discussed.

### Select “effective” filters

When the previous step is done, it is time to evaluate the filters belonging to the last layer $\left({l}^{*}\right)$ in modified structure. Obviously, the output of each layer is the integration of its filter responses. So, $\{{x}_{{l}^{*}}^{q}{\}}_{q=1}^{Q}$ can be rewritten as follows:

5 $$\{{x}_{{l}^{*}}^{q}{\}}_{q=1}^{Q}=\{{f}_{{l}^{*}}^{1},{f}_{{l}^{*}}^{2},\dots ,{f}_{{l}^{*}}^{\#{l}^{*}}{\}}_{q=1}^{Q},$$

where $\#{l}^{*}$ is the total number of filters related to ${{l}^{*}}^{th}$ layer. Employing Eq. (5), SI related to each filter or a group of filters can be computed separately. Here, the goal is to select the best group of filters that has an acceptable SI compared to the SI measured for all filters in ${{l}^{*}}$th layer. Like the previous section, the following inequality is introduced for selecting filters.

6 $$ \frac{{{\text{SI}}(\{ x_{{l^{*} }}^{q} \}_{q = 1}^{Q} ) - {\text{SI}}(\{ f_{{l^{*} }}^{{1^{*} }} ,f_{{l^{*} }}^{{2^{*} }} , \ldots ,f_{{l^{*} }}^{{m^{*} }} \}_{q = 1}^{Q} )}}{{{\text{SI}}(\{ x_{{l^{*} }}^{q} \}_{q = 1}^{Q} )}}*100 \le P_{f} \% , $$

where ${P}_{f}$ is the percentage error that can be tolerated in the effective filter selection and ${m}^{*}$ is the smallest number of best features needed to satisfy the inequality presented in (6). Due to maintaining the classifier performance, ${P}_{f}$ can be chosen up to 2%. The well-known forward selection method is used in order to choose the best filter group that satisfies Eq. (6). For selecting the first filter, the following equation should be considered.

7 $$ \begin{aligned} & i_{1}^{*} = \mathop {\arg }\limits_{n} \mathop {\max } SI(\{ f_{{l^{*} }}^{n} \}_{q = 1}^{Q} ), \\ & n \in \left\{ {1,2, \ldots ,\# l^{*} } \right\}; \\ \end{aligned} $$

After the first filter is set, it is time to find the further filters. This process should be repeated until a sufficient number of filters is chosen so that Eq. (6) becomes true. For example, in *n*th stage, the *n*th filter should be founded as follows:

8 $$ \begin{aligned} & i_{n}^{*} = \mathop {\arg }\limits_{n} \mathop {\max } {\text{SI}}(\{ f_{{l^{*} }}^{{1^{*} }} ,f_{{l^{*} }}^{{2^{*} }} , \ldots ,f_{{l^{*} }}^{n} \}_{q = 1}^{Q} ), \\ & n \in \left\{ {1,2, \ldots ,\# l^{*} } \right\} - \left\{ {i_{1}^{*} ,i_{2}^{*} , \ldots ,i_{n - 1}^{*} } \right\}; \\ \end{aligned} $$

Notably, the corresponding produced feature map is removed when a filter is eliminated. The output of the last modified layer ($${l}^{*})$$ with its optimum filters is shown as follows in the rest of the paper:

9 $$\{{\widetilde{x}}_{{l}^{*}}^{q}{\}}_{q=1}^{Q}=\{{f}_{{l}^{*}}^{{1}^{*}},{f}_{{l}^{*}}^{{2}^{*}},\dots ,{f}_{{l}^{*}}^{{n}^{*}}{\}}_{q=1}^{Q}.$$

To achieve the best set of filters of the previous layers, same process is carried out, and the best group of features of each layer is chosen.

In transfer learning, some unrelated and non-informative feature maps are probably generated since the desired dataset has not participated in the training process. Due to such feature maps, the SI of an efficient set of features could be greater than the whole features existing in the layer. Nevertheless, one cannot expect significant improvement in SI by selecting a bunch of features instead of using all of them. Therefore,

Instead of using Eq. (6), another method for terminating the feature selection process is proposed. The process will be terminated if SI does not improve considerably after the k step. The termination condition could be formulated as:

10 $$\frac{SI(\{{f}_{{l}^{*}}^{{1}^{*}},{f}_{{l}^{*}}^{{2}^{*}},\dots ,{f}_{{l}^{*}}^{{(m+k)}^{*}}{\}}_{q=1}^{Q})-SI(\{{f}_{{l}^{*}}^{{1}^{*}},{f}_{{l}^{*}}^{{2}^{*}},\dots ,{f}_{{l}^{*}}^{{m}^{*}}{\}}_{q=1}^{Q})}{SI(\{{f}_{{l}^{*}}^{{1}^{*}},{f}_{{l}^{*}}^{{2}^{*}},\dots ,{f}_{{l}^{*}}^{{(m+k)}^{*}}{\}}_{q=1}^{Q})}*100\le {P}_{f}\%,$$

### Design and train new FC structure

By removing ineffective layers and selecting effective filters, a new convolutional structure is formed. This pruned structure presenting better generalization is more compressed and accelerated. Afterward, appropriate FCLs should be developed to complete and follow the optimized filtering structure. The FC structure should be designed in such a way that it can correctly separate the formed sub-patterns. In addition, the designed network structure should have maintained the generalization provided by the filtering part because the first design principle in this paper is the size and optimality of the network. Therefore, for designing the partitioning part, SI is retaken into account for the sake of optimality.

To assess the generalization of the filtering and classification parts of the network, the SI is compared between the CLs and the FCLs. A high SI for the latest FCL compared to the latest CL indicates over-parametrization and a high sensitivity to training data, which can lead to poor performance on validation data. Therefore, an efficient scenario is when the SI for the latest CL is equivalent or similar to the SI for the latest FCL. This can guide the choice of the number of FCLs and neurons required for optimal performance.

11 $$\frac{\text{SI}(\{{\widetilde{x}}_{{l}^{*}}^{q}{\}}_{q=1}^{Q})-\text{SI}({f}_{M}(\{{\widetilde{x}}_{{l}^{*}}^{q}{\}}_{q=1}^{Q}))}{\text{SI}(\{{\widetilde{x}}_{{l}^{*}}^{q}{\}}_{q=1}^{Q})}*100\le {P}_{c}\%,$$

where ${f}_{M}(.)$ is the input–output relation of FCLs, and $M$ represents the complexity (number of FCLs and the corresponding neurons). For example, it can declare the kernel function settings in support vector machines or dense layer settings in FC structure. ${P}_{c}$ is the percentage error that can be tolerated in FCLs design. Due to maintaining the classifier performance, ${P}_{c}$ can be chosen up to 1%.

The complexity of FCLs represented by $M$ is gradually increased until the inequality in Eq. (11) is satisfied by the try and error method. The optimum complexity achieved in this process is called ${M}^{*}$.

According to CSI's high correlation with accuracy shown in section IV.A, one approach would be the usage of CSI instead of SI in designing FCLs. Moreover, the calculation of CSI is time efficient compared to SI which could be more practical in finding optimal number of neurons in an exhaustive search. Therefore, the following inequality is used in order to determine the complexity of FCLs instead of Eq. (11):

12 $$\frac{\text{CSI}(\{{\widetilde{x}}_{{l}^{*}}^{q}{\}}_{q=1}^{Q})-C\text{SI}({f}_{M}(\{{\widetilde{x}}_{{l}^{*}}^{q}{\}}_{q=1}^{Q}))}{\text{CSI}(\{{\widetilde{x}}_{{l}^{*}}^{q}{\}}_{q=1}^{Q})}*100\le {P}_{c}\text{\%},$$

## Results

In many cases, neural networks can be over-designed, with excessive capacity and parameters that can lead to over-parametrization and a loss of generalization. In this paper, we focus on detecting and addressing these redundancies and pruning them by implementing a three-step algorithm until an optimal network is achieved.

To investigate this scenario, we apply our approach to a real case study, where we comprehensively implement each of the three phases of the algorithm. In addition to this, the proposed method is compared with prior state-of-the-art methods on different benchmark networks in this section. These experiments are conducted on CIFAR-10^55^ and ImageNet^56^ datasets.

### Transfer learning by inception V3 model and cats-vs-dogs’ data

Here, an example is given to illustrate the usage of compression based on the SI in transfer learning applications. In^57^, the Inception V3 model^58^ that is originally trained on the ImageNet dataset^56^ (more than 1 million high-quality pictures belonging to 1000 classes) is used to transfer features from another dataset called “Cats-vs-dogs”. All convolutional, pooling, and other dependent layers except the dense layers are used to transfer features, and three FCLs are tuned to match the extracted features with desired labels. However, the "Cats-vs-dogs" dataset contains only 2000 training samples and 1000 validation samples belonging to two groups, making it a challenging dataset due to the small number of samples and similarities among them.

**Figure 2:**
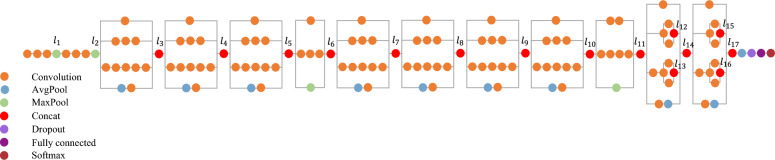
The architecture of the “Inception V3” network. The layers which are considered in layer removing procedure is highlighted with numbers (${l}_{i}, i=1, 2, \dots , 17)$.

Although pre-trained Inception V3 is used, the training process of FCLs takes approximately 35 min to achieve 89 percent of training accuracy^59^. The dimensionality of extracted features is still high ($3\times 3\times 2048$), which leads to a high number of neurons presented in FCLs. Here we want to improve or at least maintain this accuracy over a compact form of Inception V3 which is shown in Fig. 2, which is personalized for the “Cats-vs-dogs” dataset by SI. In this process, two goals are followed:

1. In transfer learning, redundant features can exist since the model is not specifically trained on the corresponding dataset. Such features can cause high dimensionality and incorrect information, making the classification problem more complex. By compressing the extracted features and removing non-informative features that do not contribute to classification, a model with fewer parameters that achieves the same or better performance can be achieved. This model can improve generalization compared to the original model, based on the parsimony principle. Generalization in transfer learning is difficult to achieve since the feature extraction part never touches the real samples.
2. One advantage of transfer learning is that using a pre-trained model is efficient. However, if tuning the last layers is time-consuming, this benefit becomes practically useless. Therefore, transfer learning may not be so interesting if the transfer model produces feature maps with high dimensionality. To address this issue, compressing the extracted features results in fewer hyperplanes needed to classify, reducing the number of neurons required in the FCLs and the time needed to learn them. This approach improves the model's generalization due to the volume reduction in the FCLs. The following describes the step-by-step compressing of the desired network by SI.

### Layer removing

**Figure 3:**
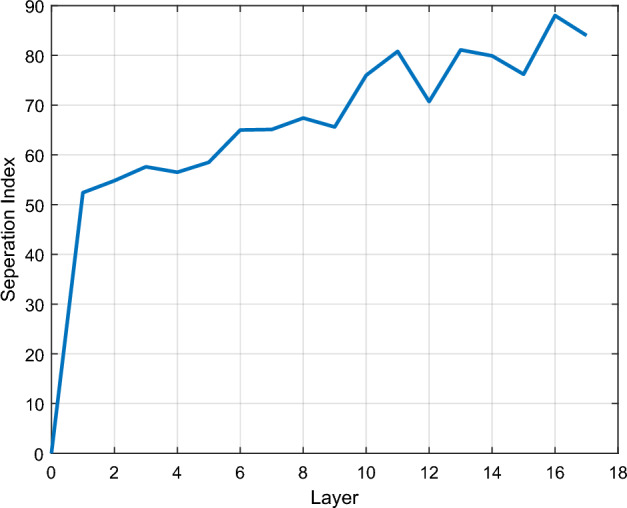
SI for dataflow of CLs belonging to the Inception V3 structure. The layer number matches the illustration in Fig. 2.

To begin with, the variation of the SI through layers is considered to investigate their effectiveness. Inception V3 has many CLs, some of which are located in blocks. Within the layers of a block, some erratic changes may appear, and the expected increasing behavior of SI may not occur. Therefore, to better evaluate feature maps produced by hidden layers, only the output of the specified blocks is considered. The location of these outputs and their corresponding layer numbers are shown in Fig. 2 (${l}_{i}, i=\text{1,2},\dots ,17)$. The computed SI at these locations is shown in Fig. 3, and SI behavior is almost increasing as we approach the end of the network. As anticipated, the maximum value of SI is reached at the end CLs, according to Fig. 3. Here, by choosing ${P}_{l}=1\%$, the smallest layer number satisfying Eq. (4) is 16.

13 $$ \begin{aligned} & {\text{SI}}(\{ x_{{l^{*} }}^{q} \}_{q = 1}^{Q} ) \ge 0.8514, \\ & {\text{SI}}(\{ x_{16}^{q} \}_{q = 1}^{Q} ) = 0.88 \to l^{*} = l_{16} ; \\ \end{aligned} $$

All unnecessary connections that are not needed to reproduce the output of ${l}_{16}$ can be truncated. The SI calculated for this layer reaches 88%, which is the maximum among all layers according to Fig. 3.

It should be mentioned that in transfer learning, SI variation is not almost increasing among the end CLs Because the network is trained with another dataset, not the desired one. Therefore, reaching maximum SI at the last few CLs is reasonable, not necessarily the last.

### Filter selection

The best combination of filters in the 16th layer that produces features with suitable SI values is needed to be found. 768 extracted 3 $\times 3$ feature maps are represented in ${16}^{th}$ layer. Here, we want to choose a subset of the best features by using the forward selection method. Table 1 shows every step of the forward selection and the achieved value for SI. As previously mentioned, SI for all features existed in 16th layer is 0.88. According to Table 1, With a sufficient number of best features, SI can be equal to the value of all features represented in 16th layer. Additionally, SI can be improved by selecting more informative features. The condition provided in Eq. (10) is used to terminate the forward selection process. By selecting $k=3$ and ${P}_{f}=0.001$, the process terminates after 34 sequential steps. At 34th step, SI reaches 91.6%, which is higher than SI for all features, and if this process continues, no improvement will be observed since all informative filters are selected approximately. Accordingly, utilizing our proposed method, the best subset of feature maps can bring more separability than all the feature maps in the layer. Finally, the outputs of these nominated filters are considered the input of FC structure, and all the other feature maps generated by discarded filters are neglected.

**Table 1:** SI measured for each group of filters in the forward selection process for cats-vs-dogs example.

Number of best features	SI
1	$SI\left({\left\{{f}_{{l}^{*}}^{{1}^{*}}\right\}}_{q=1}^{Q}\right)=0.759$
2	$SI\left({\left\{{f}_{{l}^{*}}^{{1}^{*}},{f}_{{l}^{*}}^{{2}^{*}}\right\}}_{q=1}^{Q}\right)=0.831$
3	$SI\left({\left\{{f}_{{l}^{*}}^{{1}^{*}},{f}_{{l}^{*}}^{{2}^{*}},{f}_{{l}^{*}}^{{3}^{*}}\right\}}_{q=1}^{Q}\right)=0.842$
4	$SI\left({\left\{{f}_{{l}^{*}}^{{1}^{*}},{f}_{{l}^{*}}^{{2}^{*}},{f}_{{l}^{*}}^{{3}^{*}},{f}_{{l}^{*}}^{{4}^{*}}\right\}}_{q=1}^{Q}\right)=0.850$
5	$SI\left({\left\{{f}_{{l}^{*}}^{{1}^{*}},{f}_{{l}^{*}}^{{2}^{*}},\dots ,{f}_{{l}^{*}}^{{5}^{*}}\right\}}_{q=1}^{Q}\right)=0.854$
10	$SI\left({\left\{{f}_{{l}^{*}}^{{1}^{*}},{f}_{{l}^{*}}^{{2}^{*}},\dots ,{f}_{{l}^{*}}^{{10}^{*}}\right\}}_{q=1}^{Q}\right)=0.882$
15	$SI\left({\left\{{f}_{{l}^{*}}^{{1}^{*}},{f}_{{l}^{*}}^{{2}^{*}},\dots ,{f}_{{l}^{*}}^{{15}^{*}}\right\}}_{q=1}^{Q}\right)=0.887$
20	$SI\left({\left\{{f}_{{l}^{*}}^{{1}^{*}},{f}_{{l}^{*}}^{{2}^{*}},\dots ,{f}_{{l}^{*}}^{{20}^{*}}\right\}}_{q=1}^{Q}\right)=0.894$
25	$SI\left({\left\{{f}_{{l}^{*}}^{{1}^{*}},{f}_{{l}^{*}}^{{2}^{*}},\dots ,{f}_{{l}^{*}}^{{25}^{*}}\right\}}_{q=1}^{Q}\right)=0.904$
30	$SI\left({\left\{{f}_{{l}^{*}}^{{1}^{*}},{f}_{{l}^{*}}^{{2}^{*}},\dots ,{f}_{{l}^{*}}^{{30}^{*}}\right\}}_{q=1}^{Q}\right)=0.913$
31	$SI\left({\left\{{f}_{{l}^{*}}^{{1}^{*}},{f}_{{l}^{*}}^{{2}^{*}},\dots ,{f}_{{l}^{*}}^{{31}^{*}}\right\}}_{q=1}^{Q}\right)=0.914$
32	$SI\left({\left\{{f}_{{l}^{*}}^{{1}^{*}},{f}_{{l}^{*}}^{{2}^{*}},\dots ,{f}_{{l}^{*}}^{{32}^{*}}\right\}}_{q=1}^{Q}\right)=0.915$
33	$SI\left({\left\{{f}_{{l}^{*}}^{{1}^{*}},{f}_{{l}^{*}}^{{2}^{*}},\dots ,{f}_{{l}^{*}}^{{33}^{*}}\right\}}_{q=1}^{Q}\right)=0.915$
34	$SI\left({\left\{{f}_{{l}^{*}}^{{1}^{*}},{f}_{{l}^{*}}^{{2}^{*}},\dots ,{f}_{{l}^{*}}^{{34}^{*}}\right\}}_{q=1}^{Q}\right)=0.916$
35	$SI\left({\left\{{f}_{{l}^{*}}^{{1}^{*}},{f}_{{l}^{*}}^{{2}^{*}},\dots ,{f}_{{l}^{*}}^{{35}^{*}}\right\}}_{q=1}^{Q}\right)=0.916$
36	$SI\left({\left\{{f}_{{l}^{*}}^{{1}^{*}},{f}_{{l}^{*}}^{{2}^{*}},\dots ,{f}_{{l}^{*}}^{{36}^{*}}\right\}}_{q=1}^{Q}\right)=0.915$
37	$SI\left({\left\{{f}_{{l}^{*}}^{{1}^{*}},{f}_{{l}^{*}}^{{2}^{*}},\dots ,{f}_{{l}^{*}}^{{37}^{*}}\right\}}_{q=1}^{Q}\right)=0.914$

The SI value does not improve from 34th stage.

### FC structure

The resultant output of selected filters is fed to a custom FC structure to predict labels. Considering the previous part, each input sample passes through the compressed filtering structure, and a $3\times 3\times 34$ matrix is formed as output which can be reformed to an array with 306 features. Two hidden dense layers are used and learned for partitioning these filtered samples like in the previous example. Here, Eq. (12) is rewritten based on this case as follows:

14 $$\frac{FSI\left({\left\{{f}_{16}^{{1}^{*}},{f}_{16}^{{2}^{*}},\dots ,{f}_{16}^{{34}^{*}}\right\}}_{q=1}^{Q}\right)}{FSI\left({\left\{{f}_{16}^{{1}^{*}},{f}_{16}^{{2}^{*}},\dots ,{f}_{16}^{{34}^{*}}\right\}}_{q=1}^{Q}\right)}-\frac{FSI({f}_{M}({\left\{{f}_{16}^{{1}^{*}},{f}_{16}^{{2}^{*}},\dots ,{f}_{16}^{{34}^{*}}\right\}}_{q=1}^{Q}))}{FSI({\left\{{f}_{16}^{{1}^{*}},{f}_{16}^{{2}^{*}},\dots ,{f}_{16}^{{34}^{*}}\right\}}_{q=1}^{Q})}*100\le {P}_{c}\%,$$

The number of neurons in each FC is determined by comparing CSI at the beginning and end of the structure. $\text{CSI}({\left\{{f}_{{l}^{*}}^{{1}^{*}},{f}_{{l}^{*}}^{{2}^{*}},\dots ,{f}_{{l}^{*}}^{{34}^{*}}\right\}}_{q=1}^{Q})$ which is the computed CSI at the beginning of structure is computed and equal to 0.926. According to Eq. (14), by selecting ${P}_{c}=1\%$, the hyperparameter of the dense layer can be computed as follows:

15 $$\frac{0.926-F\text{SI}({f}_{{M}^{*}}({\left\{{f}_{{l}^{*}}^{{1}^{*}},{f}_{{l}^{*}}^{{2}^{*}},\dots ,{f}_{{l}^{*}}^{{34}^{*}}\right\}}_{q=1}^{Q}))}{0.926}\le 0.01,\to F\text{SI}\left({f}_{{M}^{*}}\left({\left\{{f}_{{l}^{*}}^{{1}^{*}},{f}_{{l}^{*}}^{{2}^{*}},\dots ,{f}_{{l}^{*}}^{{34}^{*}}\right\}}_{q=1}^{Q}\right)\right)\ge 0.9167$$

Additionally, all CSI values and accuracy in each step of this process are reported in Table 2 to determine the complexity of the dense layer. Therefore, if the complexity is assumed to be a vector of two parameters as shown in Eq. (16), the simplest complexity satisfying Eq. (15) is ${M}^{*}=\left[11 11\right]$.

**Table 2:** CSI and accuracy of train and test data for different complexity for Cats-vs-dogs example.

	Number of neurons (M)	CSI of last FC layer (Train data)	CSI of last FC layer (Test data)	Accuracy (Train data)	Accuracy (test data)
1	[1 1]	0.824	0.792	0.843	0.803
2	[3 3]	0.8445	0.835	0.861	0.837
3	[5 5]	0.884	0.877	0.892	0.891
4	[7 7]	0.906	0.912	0.907	0.912
5	[9 9]	0.907	0.918	0.90	0.916
6	[11 11]	0.918	0.911	0.92	0.922
7	[13 13]	0.928	0.924	0.925	0.924
8	[15 15]	0.928	0.92	0.929	0.921
9	[17 17]	0.931	0.923	0.928	0.923
10	[19 19]	0.932	0.916	0.9305	0.916
11	[21 21]	0.945	0.916	0.948	0.915
12	[30 30]	0.9775	0.9199	0.977	0.898
13	[40 40]	0.9811	0.898	0.9813	0.897
14	[50 50]	0.9832	0.899	0.9835	0.898

It shows the high correlation between CSI and accuracy both in train and test data.

16 $$M=\left[\text{neurons of }1\text{st hidden layer neurons of }2\text{nd hidden layer}\right]$$

Since the design of the entire compressed network is finished, it can be compared with the original Inception V3 in terms of volume, memory, and performance. Regarding compression, six CLs are discarded, and the size of the input of the FC layers is reduced to $3\times 3\times 34$ instead of $3\times 3\times 2048$. The output of all these filters is aggregated into a single array with 306 elements, an input to the upcoming designed FC structure. The model complexity increases gradually until the CSI of the last FC layer reaches the CSI of the nominated subset of filters. Finally, in Table 3, the advantages of compressing the Inception V3 are summarized. Compared to the results presented in^59^, nearly half of all network parameters are discarded. Surprisingly, 99% of FC parameters are put aside thanks to the proposed feature selection method. In addition, the accuracy of the network is slightly increased.

**Table 3:** Comparison between Modified Inception V3^59^ and the compressed network resulted from the proposed algorithm.

Network	Modified Inception V3	Compressed Inception V3
All parameters	40,679,200	19,074,969
Convolutional parameters	21,802,784	19,071,460
Fully Connected parameters	18,876,416	3,509
Training accuracy (%)	89.10	92.00
Test accuracy (%)	91.56	92.20

In the following, the compression method with SI is compared with state-of-the-art works in several cases. Firstly, it is checked whether each method needs retraining. Additionally, the ratio of parameters pruned in the compressed network is reported in each example. Also, the reduced FLOPs are reported to show how much faster the compact model is than the base model. Finally, the accuracy of the compressed models is shown for comparison purposes.

### Results on CIFAR-10

Various approaches can be found in the literature in compressing CNN. Here, a comparison has been made between the proposed method and some other compressing methods in classifying some popular models trained by the CIFAR-10 dataset, i.e., VGG-16, GoogLeNet, DenseNet-40 and ResNet-56. A variant of VGG presented in^25^ is used in this comparison. For GoogLeNet, we take a branch of the base one in which the final output class is fitted to CIFAR-10. Also, the DenseNet-40 comprises 40 layers with a growth rate of 12.

*VGG16*. Here, a comparison with other state-of-the-art compressing methods such as PFEC^25^, VP^60^, SS^61^, EIC^62^, GAL^63^, CP^64^, and HRFM^65^ is made and reported in Table 4. The result shows that our method performs better in pruning parameters and FLOPS reduction. The obtained results also surpass PFEC^25^, VP^60^, SS^61^, and HRFM^65^, in which the network needs to be retrained. The result reported in HRFM^65^ and PPCA^66^ is better than the proposed method in reducing parameters (88.2% vs. 87.5% and 92.7% vs. 87.5%) and FLOPs (76.5% vs. 76% and 92.7% vs 76%). However, the accuracy becomes noticeably lower than the result obtained by the proposed method (91.23% vs. 93.49%). GA^67^ achieved promising memory reduction rates using genetic algorithms, such as a 90% reduction in memory when compressing VGG-16 on CIFAR-10. However, the accuracy of the resulting compressed model was considerably lower than that of state-of-the-art methods, at only 79.67%.

**Table 4:** Comparison with the prior art of compressing VGG-16 trained on CIFAR-10.

Compressing method	Retraining needed	FLOPs	Pruned	Accuracy
VGG16-base	–	313.7M (0.0%)	0.0%	94.04%
PFEC^25^	Yes	206M (34.3%)	63.3%	93.40%
VP^60^	Yes	190M (39.1%)	73.4%	93.18%
SS^61^	Yes	183.13M (41.6%)	73.2%	93.02%
EIC^62^	No	177.27M (43.4%)	77.6%	92.49%
GAL-0.05^63^	No	189.49M (39.6%)	77.2%	92.03%
CP^64^	No	107.58M (65.1%)	77.6%	92.03%
HRFM^65^	Yes	108.61M (65.3%)	82.1%	92.34%
GAL-0.1^63^	No	171.89M (45.2%)	82.2%	90.73%
Proposed method	**No**	**75.6M (76.0%)**	**87.5%**	**93.49% ± 0.002**
HRFM^65^	Yes	73.70M (76.5%)	88.2%	91.23%
PPCA^66^	Yes	22.9M (92.7%)	92.7%	92.61%
GA^67^	No	N/A	87.5%	79.67%

Significant values are in bold.

*GoogLeNet*. Again, the proposed method shows its ability to accelerate another network, i.e., GoogLeNet. According to Table 5, a considerable reduction in FLOPs and parameters (74.4% and 77.6%, respectively) occurs since the model's accuracy is barely changed (95.05% vs. 95.00%). The result clearly demonstrates that the SI is a powerful tool to find efficient filters and accelerate the network without losing accuracy. It also surpasses the adaptive importance-based methods such as GAL-Apo^68^ and GAL-0.05^64^ in terms of accuracy (95.00% vs. 92.11% by GAL-Apo and 93.93% by GAL-0.05). After the SI identifies the efficient filters, only a simple tuning of an FC structure is needed to map the output of the selected filters to labels. This process is much faster than the pruning method such as PFEC^25^ and HRFM^65^ that prune inefficient filters by retraining the whole structure.

**Table 5:** Comparison with the prior art of compressing GoogLeNet trained on CIFAR-10.

Compressing method	Retraining needed	FLOPS	Pruned	Accuracy
GoogleNet-base	–	1.52B (0.0%)	0.0%	95.05%
PFEC^25^	Yes	1.0B (32.9%)	42.9%	94.54%
HRFM^65^	Yes	0.69B (54.9%)	55.4%	94.53%
GAL-Apo^68^	No	0.76B (50.0%)	53.7%	92.11%
GAL-0.05^64^	No	0.94B (38.2%)	49.3%	93.93%
HRFM^65^	Yes	0.45B (70.4%)	69.8%	94.07%
Proposed method	**No**	**0.39B (74.4%)**	**77.6%**	**95.01% ± 0.0001**

Significant values are in bold.

*DenseNet*. For DenseNet, the accuracy only drops 0.04% using the proposed method according to Table 6. Also, a considerable space is saved by removing 78.8% of parameters that the SI identifies as redundant parameters. Also, the computation is boosted in the compressed network by reducing 76.1% FLOPs. These reductions have taken place thanks to the SI, despite the fact that DenseNet itself is a compact network. Given that the SI is calculated in a supervised manner, there is no need to retrain the network during or after the compression process. In addition, only inefficient filters are pruned, and the model's accuracy does not reduce considerably. As a result, it can challenge compressing methods needed retraining, such as ECNS^69^ and HRFM^65^.

**Table 6:** Comparison with the prior art of compressing DenseNet-40 on trained CIFAR-10.

Compressing method	Retraining needed	FLOPS	Pruned	Accuracy
DenseNet-base	–	0.29B (0.0%)	0.0%	94.22%
ECNS^69^	Yes	0.12B (58.3%)	67.2%	94.35%
HRFM^65^	Yes	0.11B (61.8%)	55.1%	94.53%
GAL-0.05^64^	No	0.13B (55.6%)	57.9%	92.11%
VP^60^	No	0.16B (45.8%)	60.7%	93.16%
Proposed method	**No**	**0.07B (76.1%)**	**78.8%**	**94.15% ± 0.0007**

Significant values are in bold.

*ResNet*. The proposed method achieves the best result among all the prior arts in Compressing ResNet56 structure and the results are shown in Table 7. The ResNet structure comprises many identity maps which are identified to be not informative by SI evaluation. Our algorithm based on SI pruned 57.8% of parameters and improved speed of the network by 58.3%. Hopefully, no training phase is needed after pruning and only a little tuning of fully connected layers is needed. The performance of the compressed model is approximately equal to the base structure and the accuracy is decreased only 0.03%.

**Table 7:** Comparison with the prior art of compressing ResNet-56 trained on CIFAR-10.

Compressing method	Retraining needed	FLOPS	Pruned	Accuracy
ResNet-base	–	125.49M (0.0%)	0.0%	93.26%
SFP^70^	No	59.48M (52.6%)	50.6%	91.93%
GAL^63^	Yes	64.62M (48.5%)	44.8%	92.74%
FPGM^71^	Yes	59.48M (52.6%)	50.6%	93.16%
HRFM^65^	Yes	62.72M (50.0%)	42.4%	93.17%
SCOP^72^	Yes	55.21M (56.0%)	56.3%	93.20%
RUFP^73^	Yes	53.7M (57.7%)	56.8%	93.17%
Proposed method	**No**	**52.32M (58.3%)**	**57.8%**	**93.22% ± 0.01**

Significant values are in bold.

### Results on ImageNet

Some experiments are also carried out on challenging ImageNet dataset. Considering the high dimensionality and scale of ImageNet, only the ResNet-50 architecture has been considered for compression, and the obtained results have been compared with other state-of-the-art methods in Table 8.

**Table 8:** Comparison with the prior art of compressing ResNet-50 trained on ImageNet (ILSVRC-2012).

Compressing method	Retraining needed	FLOPS	Pruned	Accuracy
ResNet-base	–	4.09B (0.0%)	0.0%	76.20%
SFP^70^	No	2.38B (41.8%)	N/A	74.61%
GAL^63^	Yes	2.33B (43%)	16.9%	71.95%
FPGM^71^	Yes	2.36B (42.2%)	37.5%	75.59%
HRFM ^65^	Yes	2.3B (43.7%)	36.7%	74.98%
SCOP^72^	Yes	2.24B (45.3%)	42.8%	75.95%
SCWC^74^	Yes	2.29B (44.1%)	43.8%	76.02%
APOZ^75^	Yes	1.99B (51.3%)	52.2%	72.68%
Proposed method	**No**	**2.21B (46.0%)**	**44.7%**	**76.06% ± 0.03**

Significant values are in bold.

Our proposed compression method achieves an accuracy for the compressed model that is almost as high as the accuracy of the base model, and with no need for model retraining. Moreover, the resulting accuracy loss is minimal compared to other approaches such as SCWC^74^ and SCOP^72^. While the APOZ^75^ method reports the best pruning rate and reduced FLOPs among all the methods, the accuracy decreases by 3.56%, which is not desirable compared to other methods.

## Discussion

### Comparison between the proposed method and traditional method of designing fully connected layers

SI and CSI can be used to design the hyper-parameters of the system. The method used for designing FC layers is not limited to this paper and can be used for any FC structure. This method avoids the over-parametrization situation and forces the system to keep generalization.

**Figure 4:**
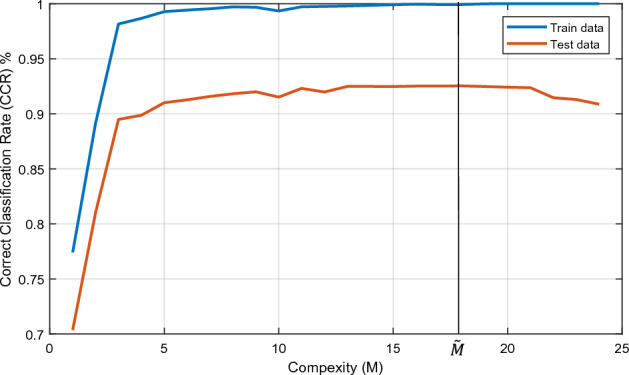
Training and testing accuracy for CIFAR-10, trained on a compressed VGG-16 model. This figure illustrates the procedure for selecting hyper-parameters by observing fluctuations in test accuracy.

One approved traditional approach for maintaining generalization in the training process is to divide data into train and validation groups. In this method, the termination condition of the training process is determined by validation data. If the accuracy of validation data does not improve through increasing complexity, the training process should be terminated. The setting that causes termination is reported as the optimum value for hyper-parameters of the network, which controls the sensitivity of the network to train data. accuracy for train and test data according to the complexity of the system (denoted by $M$) is illustrated in Fig. 4. Also, the location of optimum complexity (denoted by $\widetilde{M}$) which is computed by the mentioned traditional approach has appeared in this figure.

The comparison results between the above method and the proposed method are reported in Table 9. As it can be seen, there is not much difference in test accuracy even though the number of neurons in the proposed method is less than the traditional one.

**Table 9:** The comparison between the traditional and proposed methods in selecting hyper-parameters in designing a fully connected structure.

The approach of selecting $\text{M}$	The resultant value for $\text{M}$	Test accuracy (%)
Traditional method	$\widetilde{M}=[140 130]$	93.54
Proposed method	${M}^{*}=[110 110]$	93.49

The accuracy achieved by the proposed method appears to be comparable to that of the traditional method, despite the proposed method utilizing a lower number of network neurons.

The main advantage of the proposed method is that no additional data is used to control the network's generalization. By measuring the CSI of training data itself, the over-parametrization situation can be detected. Comparison of CSI measured at last CL and last FCL for training data can control whether the sufficient number of decision surfaces is correctly specified or not. Adding more layers and neurons in the learning process allows the CSI of the last FCL to excel CSI of the last CL. However, this could be a warning that the number of decision surfaces is too much and over-parametrization issue is probable. This excellence can be instrumental when there is limited access to another group of data or training data is inadequate, and all samples are needed to participate in the learning process. In the transfer learning problem, once again, this method is successfully used to design FC structure and prevents the structure from having redundant complexities.

### The impact of ${{P}}_{{l}}$ and ${{P}}_{{f}}$

As describe in Section III.A and III.B, ${P}_{l}$ and ${P}_{f}$ are threshold values used in pruning layer and filters of the base network. In the compressing algorithm, a trade-off between compression rate and accuracy can be achieved by adjusting these parameters. The impact of ${P}_{l}$ is much greater than ${P}_{f}$ because changing ${P}_{l}$ can result in pruning a significant proportion of a layer's parameters, whereas changing ${P}_{f}$ has a lesser impact on the pruning of network elements.

The impact of these parameters on compressing VGG-16 on CIFAR-10 was analyzed, and the resulting accuracy and pruning rate was recorded in Tables 10 and 11, respectively. By increasing each parameter, the accuracy becomes lower since the number of pruned parameters increases. In each column of Table 10, ${P}_{l}$ decreased from top to bottom. However, in the last rows of each column, the rate of decline in accuracy was higher as the pruned parameters were more important. The effect of ${P}_{f}$ on accuracy loss was found to be minor, and it can be adjusted for a more precise result. For each cell of this table the entire algorithm of compressing is carried out.

**Table 10:** Accuracy (%) of compressed VGG-16 on CIFAR-10 with different values of ${P}_{l}$ and ${P}_{f}$.

${P}_{l}$/${P}_{f}(\%)$	0	0.5	1	2	3
0	94.04	94.08	94.06	94.02	93.94
1	93.87	93.86	93.86	93.83	93.81
2	93.49	93.49	93.47	93.44	93.41
3	92.87	92.84	92.83	92.81	92.81
4	90.04	90.01	89.95	89.92	89.88
5	86.99	86.93	86.89	86.82	86.78

**Table 11:** Pruning rate (%) of compressed VGG-16 on CIFAR-10 with different values of ${P}_{l}$ and ${P}_{f}$.

${P}_{l}$/${P}_{f}$(%)	0	0.5	1	2	3
0	0.0	10.84	10.88	10.89	10.90
1	71.31	71.32	71.32	71.37	71.38
2	87.47	87.5	87.53	87.57	87.58
3	89.32	89.39	89.40	89.43	89.43
4	90.11	90.13	90.15	90.15	90.18
5	90.28	90.31	90.32	90.34	90.34

As illustrated in Table 11, when ${P}_{l}=0\%$ and ${P}_{f}=0\%$, no parameter is pruned in compressing algorithm since there is no allowance for accuracy loss. The pruning process starts by increasing each parameter. Considering last row of Table 11, it is observed that the rate of pruning changes very slowly by increasing ${P}_{f}$. However, the corresponding row in Table 10 shows that the accuracy changes at a faster pace. This is because the network parameters have been pruned to such an extent that removing even a small number of parameters results in a significant decrease in accuracy. By monitoring changes in ${P}_{l}$ and ${P}_{f}$, one can determine how the threshold value should be adjusted to prevent a significant drop in accuracy. It is logically beneficial to freeze the ${P}_{f}$ and adjust ${P}_{l}$ at first, and then adjust ${P}_{f}$ for more desirable result.

### Time and computational analysis of the proposed algorithm

The algorithm was executed on a system featuring an Intel 12700K CPU, 32 gigabytes of DDR4 RAM, and a NVIDIA RTX 3090 GPU for enhanced computational performance. Our implementation code of the convolutional neural network compression method using the Separation Index is in Python utilizing the PyTorch library. The code is optimized for efficiency, with computationally intensive tasks executed in parallel on the GPU.

The computation time of Separation Index is very effective in duration of the proposed algorithm implementation. Two influential factors in computation of Separation Index are the number of data and its dimension.

To mitigate this computational overhead, we have strategically chosen batch sizes tailored to the specific datasets utilized. For CIFAR-10, a batch size of 5000 samples is employed, while for ImageNet, a batch size of 20,000 samples is deemed optimal. By averaging the Separation Index value of these batch sizes, we aim to strike a balance between computational efficiency and accurate evaluation.

Moreover, to provide insight into the method's efficiency, we conducted experiments to measure the time taken for each phase. The summarized results are presented in Table 12.

**Table 12:** Time analysis of the proposed compression algorithm on different experiments, including the duration of convolutional layer pruning and fully connected layer tuning, reported and compared across various datasets and network architectures.

Dataset	Network	Convolutional layer pruning duration (second)	Fully connected layer tuning duration (second)
CIFAR-10	VGG-16	1643.34	54.67
CIFAR-10	GoogLeNet	561.76	21.11
CIFAR-10	DenseNet40	383.12	26.79
CIFAR-10	ResNet-56	364.70	63.42
ImageNet	ResNet-50	2843.55	1094.34

It is noteworthy that VGG-16 exhibits a lengthier duration for convolutional layer pruning due to its higher pruning rate compared to the other networks. Conversely, the tuning phase duration for fully connected layers remains relatively consistent across the CIFAR-10 networks, as all fully connected structures entail relatively minimal design complexities.

When compressing ResNet-50 trained on the ImageNet dataset, we encounter a higher volume of feature maps due to the extensive nature of this dataset, which comprises over a million images. Consequently, the computation of the SI necessitates numerous iterations, and the process of convolutional pruning demands a substantial amount of time compared to networks trained on the CIFAR-10 dataset. Furthermore, given that the ImageNet dataset encompasses 1000 distinct classes, the design requires a dense fully connected structure, leading to additional time consumption during tuning processes.

SI reveals each element's competence, separation power, and filtering contribution. In addition to having the advantages of pruning methods, the proposed compressing method also has other privileges and advantages that can be mentioned here:

1. While some filters are pruning using the proposed algorithm, the residual efficient routes from input to output remain intact. Therefore, once pruning is carried out, the model does not require training from the scratch. This is particularly advantageous in transfer learning scenarios where users may not have adequate processing resources to retrain the model. Our method not only prunes non-informative filters but also identifies filters that can effectively segregate different classes, improving the model's generalization ability.
2. We have compared our method with prior state-of-the-art methods, particularly those that require training from scratch, and found that our compression with SI can perform even better in terms of FLOP reduction and pruning rate.
3. A new method is also introduced to tune the hyper parameters in designing fully connected layers based on Center-based Separation Index. This method can be used in the following scenarios. Firstly, after the convolutional layers are compressed and non-effective filters are pruned, a structure containing fully connected layers should be designed compatible to the compressed convolutional layers. The neurons number related to these fully connected layers can be determined using Center-based Separation Index according to the algorithm discussed in section III.C. In addition, in transfer learning problems, after the best set of features generated by convolutional layers are chosen based on Separation Index, the number of neurons of fully connected layers can be computed using Center-based Separation Index. Center-based Separation Index controls number of neurons to avoid system from losing generalization and over-parametrization.
4. The Separation Index can evaluate a single filter, a block of filters or even an entire layer at the same time. This ability accelerates the compressing algorithm especially at the early stages of the process since a large group of elements can be evaluated and pruned at once.

## Conclusion

An overview was performed on a geometric parameter named Separation Index, which can be used to evaluate different elements of a CNN. An extended *Center-based* Separation Index with the same characteristics and average geometric viewpoint is introduced, having a better correlation with the accuracy. An algorithm based on the Separation Index was developed to compress the filtering part of CNN so that the extra convolutional layers were removed and the filters that performed best were selected as the chosen combination. Another method based on the Center-based Separation Index was proposed to determine the hyper-parameters of fully connected layers so that the generalization of the system would be achieved. Overall, some efforts have been made to present a comprehensive compressing algorithm with minimum parametric design besides maintaining efficiency not only in filtering but in partitioning as well. The proposed method provided in three straightforward consecutive steps is applied on the CIFAR-10 and ImageNet dataset. The comparison results with state-of-the-art methods seem promising. Also, an illustrative example of compressing the Inception V3 network by the proposed method is given to classify the "Cats-vs-dogs" dataset.

## Data Availability

The datasets used in this study are readily accessible for research purposes. The specific sources and download links for each dataset are as follows: (1) Cats-vs-dogs dataset: This dataset can be obtained from Kaggle’s “Dogs vs. Cats” competition, available at Dogs vs. Cats | Kaggle. (2) CIFAR-10 dataset: The CIFAR-10 dataset is accessible through the University of Toronto's official website at https://www.cs.toronto.edu/~kriz/cifar.html. (3) Image-Net dataset: The Image-Net dataset can be downloaded from the Image-Net website, accessible at https://www.image-net.org/.
